# NagX, a periplasmic β-*N*-acetylglucosaminidase, contributes to NagZ-independent β-lactamase expression in *Stenotrophomonas maltophilia*

**DOI:** 10.1128/aac.00243-26

**Published:** 2026-07-16

**Authors:** En-Wei Hu, Po-Cheng Kuo, Li-Hua Li, Chen-Syuan Yen, Cheng-Chih Hsu, Tsuey-Ching Yang

**Affiliations:** 1 Department of Biotechnology and Laboratory Science in Medicine, National Yang Ming Chiao Tung University 548277, Taipei, Taiwan; 2 Department of Chemistry, National Taiwan University 33561, Taipei, Taiwan; 3 Department of Pathology and Laboratory Medicine, Taipei Veterans General Hospital 46615, Taipei, Taiwan; 4 School of Medical Laboratory Science and Biotechnology, College of Medical Science and Technology, Taipei Medical University 38032, Taipei, Taiwan; Shionogi Inc., Florham Park, New Jersey, USA

**Keywords:** β-lactam resistance, β-lactamase, β-*N*-acetylglucosaminidase, muropeptides, *Stenotrophomonas maltophilia*

## Abstract

Beta-lactam resistance in *Stenotrophomonas maltophilia* is primarily mediated by the expression of L1 and L2 β-lactamases. L1/L2 expression is closely linked to peptidoglycan recycling through the AmpNG-NagZ-AmpR pathway, in which NagZ plays a dominant role for L1/L2 expression. Although a NagZ-independent β-lactamase expression has been suggested, its molecular basis remains unclear. Here, we identify NagX, a periplasmic β-*N*-acetylglucosaminidase, as a key contributor to the NagZ-independent β-lactamase pathway. Using *S. maltophilia* KJ and its derived *nagZ* in-frame deletion mutant, KJΔNagZ, we confirm the presence of NagZ-independent β-lactamase expression and an additional β-*N*-acetylglucosaminidase in addition to NagZ. Determination of β-lactamase and β-*N*-acetylglucosaminidase activities in various engineered strains of *S. maltophilia* KJ demonstrated that NagX is a periplasmic β-*N*-acetylglucosaminidase and drives NagZ-independent β-lactamase expression in an AmpNG- and AmpR-dependent manner. To reveal how NagX processes the released peptidoglycan sacculus, the periplasmic muropeptides profile of wild-type KJ, KJΔAmpNG (an *ampNG* mutant), and KJΔAmpNGΔNagX (an *ampNG* and *nagX* double mutant) were determined by LC-MS/MS. The results demonstrated that NagX can remove the *N*-acetylglucosamine (GlcNAc) moiety from the GlcNAc-anhMurNAc-peptide and GlcNAc-anhMurNAc, yielding anhMurNAc-peptide and anhMurNAc, respectively. AmpNG permease system shuttled GlcNAc-anhMurNAc-peptide, GlcNAc-anhMurNAc, and anhMurNAc-peptide from periplasm to cytosol. Then, the anhMurNAc-peptide served as activator ligands that cooperate with AmpR to induce β-lactamase expression. Our findings uncover a novel NagX-AmpNG-AmpR circuit underlying NagZ-independent β-lactamase expression in *S. maltophilia*.

## INTRODUCTION

**Fig 1:**
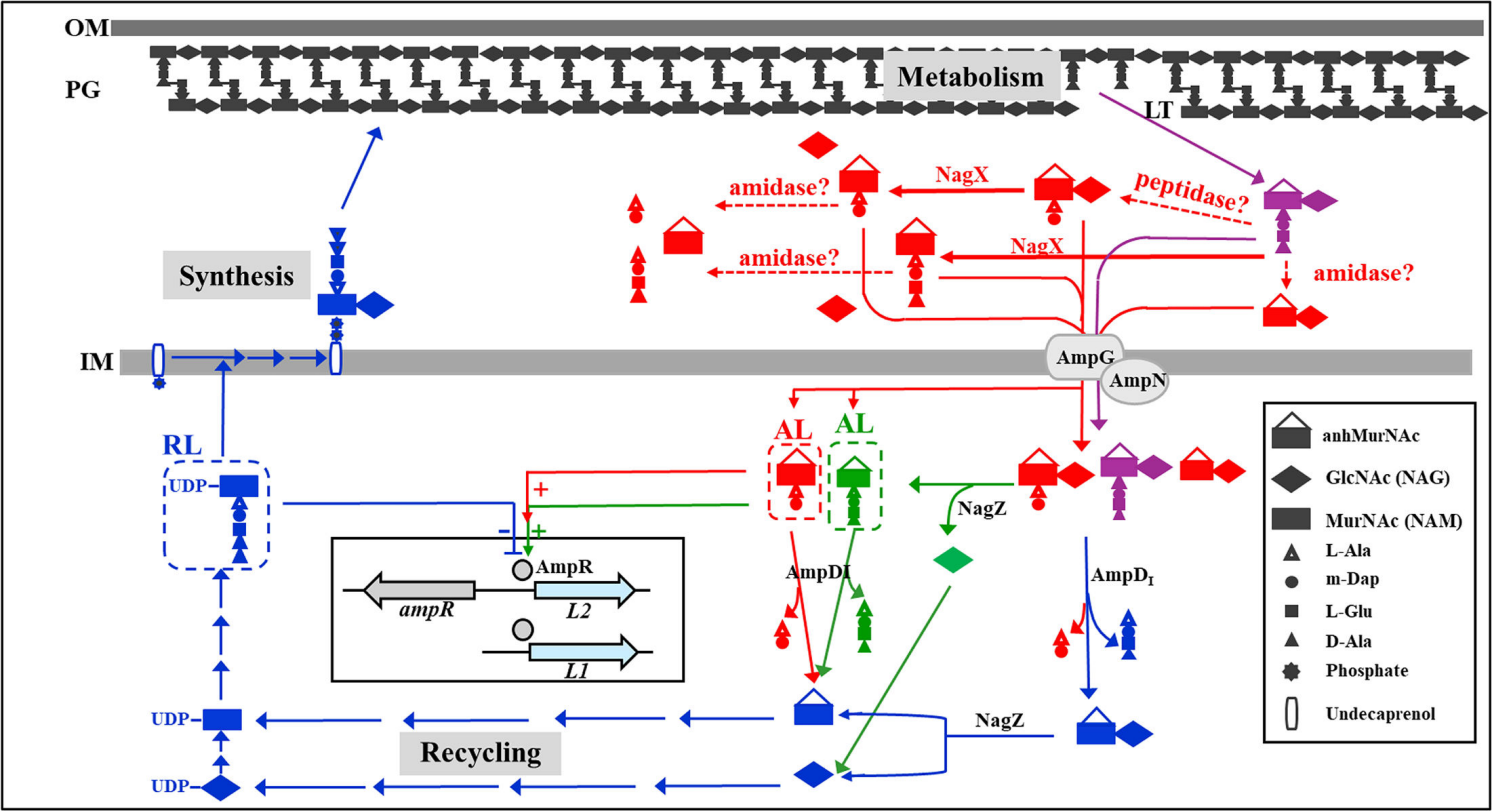
Interconnection between PG homeostasis and L1/L2 expression in *Stenotrophomonas maltophilia*. Arrows and symbols in purple, blue, and green represent the established mechanisms, whereas red highlights novel findings from this study. The dashed arrows and question marks indicate the speculated enzymes involved in the hydrolytic processes. During PG turnover, GlcNAc-anhMurNAc-tetrapeptide (M4N) is generated by lytic transglycosylases and transported into the cytoplasm via the AmpNG permease system (marked in purple). Imported M4N is mainly processed by AmpD_I_ to yield GlcNAc-anhMurNAc (M0N) and stem peptides. M0N is further hydrolyzed by NagZ, releasing GlcNAc and anhMurNAc (U0N). GlcNAc, U0N, and peptide stems are recycled into UDP-MurNAc-pentapeptide that represses L1/L2 expression (marked in blue). In the *ampD_I_* mutant, excess M4N is hydrolyzed by NagZ to generate GlcNAc and anhMurNAc-tetrapeptide (U4N), which functions as an activator ligand (AL) of L1/L2 expression (marked in green). In addition to M4N, anhMurNAc-dipeptide (M2N) is generated in the periplasm during PG turnover. There, NagX processes M4N and M2N to yield U4N, U2N, and GlcNAc. M4N, M2N, U4N, U2N, and M0N are then imported into the cytoplasm via AmpNG. When M4N and M2N are further processed by NagZ in the cytoplasm, the resulting U4N and U2N may act as ALs for L1/L2 expression (marked in red).

Peptidoglycan (PG) is an essential component that helps bacteria maintain cell shape and protects them from environmental stress (1). It undergoes continuous remodeling in response to bacterial growth and changing conditions (2). PG is composed of repeating units of monomers, each consisting of β-1,4-linked *N*-acetylglucosamine (GlcNAc) and *N*-acetylmuramic acid (MurNAc) appended with a pentapeptide stem. PG monomers are polymerized by glycosyltransferases, which catalyze the formation of linear glycan chains, and transpeptidases, which catalyze peptide cross-linking. During bacterial growth, pre-existing PG is excised from the cell wall by periplasmic enzymes, mainly lytic transglycosylases (LTs). The degraded muropeptides are then imported into the cytoplasm by specific inner membrane transporters and reused for PG monomer synthesis through the PG recycling pathway (3–6) (marked in purple in Fig. 1). PG monomers are synthesized in the cytoplasm as UDP-linked precursors, attached to a membrane-bound undecaprenyl phosphate, and flipped into the periplasm (7). In the periplasm, penicillin-binding proteins (PBPs) catalyze the incorporation of new PG monomers into the pre-existing PG (8) (marked in blue in Fig. 1).

Given the uniqueness and importance of PG for bacteria, inhibitors of PG synthesis have become some of the most effective classes of antibiotics, including β-lactams and glycopeptides (9). Beta-lactam antibiotics, which mimic the _D_-Ala-_D_-Ala moiety of PG monomers, can covalently bind to the serine active site of PBPs, thereby disrupting PG synthesis (10). Due to their widespread use, β-lactam resistance has emerged rapidly. Mechanisms of β-lactam resistance include production of β-lactamases, reduced affinity between β-lactams and PBPs, decreased outer membrane permeability, and increased efflux pump activity (11). Among these, the inducible production of chromosomally encoded β-lactamase is a common mechanism by which Gram-negative bacteria cope with β-lactam stress—for example, AmpC in *Enterobacter cloacae*, *Citrobacter freundii*, and *Pseudomonas aeruginosa*, and L1 and L2 in *Stenotrophomonas maltophilia* (12–14).

*Stenotrophomonas maltophilia*, a ubiquitous environmental microorganism, is responsible for severe nosocomial infections in immunocompromised patients and chronic lung infections in patients with cystic fibrosis (15). *S. maltophilia* is intrinsically resistant to many antibiotics, making its infections particularly difficult to treat. The genome of *S. maltophilia* encodes numerous antibiotic resistance determinants, including β-lactamases, aminoglycoside-modification enzymes, Qnr-type quinolone resistance cassettes, and efflux pumps (16). β-lactam resistance in *S. maltophilia* mainly arises from the inducible expression of chromosomally encoded L1 and L2 β-lactamases, which belong to class B and class A groups, respectively (14). Due to their broad substrate spectrum, L1 and L2 render *S. maltophilia* resistant to nearly all clinically used β-lactams (17). Similar to the AmpC model of *P. aeruginosa* (18), inducible expression of L1 and L2 is closely linked to PG recycling and regulated by AmpR, a LysR-type transcriptional regulator (14, 19). The *ampR* and *L2* genes are located adjacently and divergently transcribed, forming a divergon module. The regulatory role of AmpR in β-lactamase expression depends on the types of ligands it binds. Figure 1 depicts the PG recycling pathway and the inducible expression of L1/L2. In this article, muropeptide structures are described using a three-character abbreviated notation. The first letter denotes the sugar moiety: M represents a PG monomer containing GlcNAc and MurNAc, whereas U indicates MurNAc only. The middle digit corresponds to the number of amino acids in the stem peptide. The final letter, N, indicates the presence of an anhydro bond in MurNAc. During normal bacterial growth, the periplasmic LT-generated product GlcNAc-β-anhMurNAc-tetrapeptide (M4N) is transported into cytoplasm through the inner membrane AmpNG permease system (20) and mainly processed by the anhydro-*N*-acetyl-muramyl-l-Ala amidase AmpD_I_ (21), producing GlcNAc-anhMurNAc (M0N) and peptides (marked in purple in Fig. 1). M0N is then further hydrolyzed by the β-*N*-acetylglucosaminidase NagZ (22), which removes the GlcNAc moiety to yield GlcNAc and anhMurNAc (U0N). GlcNAc, U0N, and peptide stems are recycled to form UDP-MurNAc-pentapeptide. UDP-MurNAc-pentapeptide can serve both as the precursor for PG synthesis and as a repressor ligand (RL) that mediates L1/L2 repression (marked in blue in Fig. 1). In an *ampD_I_* mutant, AmpNG-imported M4N is hydrolyzed by NagZ, generating GlcNAc and anhMurNA-tetrapeptide (U4N). U4N acts as an activator ligand (AL) for L1/L2 induction (marked in green in Fig. 1). Thus, the basal β-lactamase activity of the *ampD_I_* mutant is derepressed (21).

Based on the well-established AmpC or L1/L2 induction models, ALs and RLs are generated through cytoplasmic PG recycling enzymes, with NagZ being essential for AL generation (23). It has been proposed that inactivation of *nagZ* can prevent and even revert β-lactam resistance (24–27), making NagZ a potential therapeutic target. However, a NagZ-independent pathway for β-lactamase expression has been observed in *S. maltophilia* and *E. cloacae* complex (22, 28, 29), though its exact mechanism remains unclear. In this study, we aimed to identify the factors involved in NagZ-independent β-lactamase expression in *S. maltophilia*. We discovered that NagX, a periplasmic β-*N*-acetylhexosaminidase, participates in this circuit. Our results reveal a unique dynamic of AL production for L1/L2 induction in *S. maltophilia*, in which both periplasmic NagX and cytoplasmic NagZ act in coordination.

## RESULTS

### *nagZ* mutant of *S. maltophilia* displays a NagZ-independent β-lactamase expression

**Fig 2:**
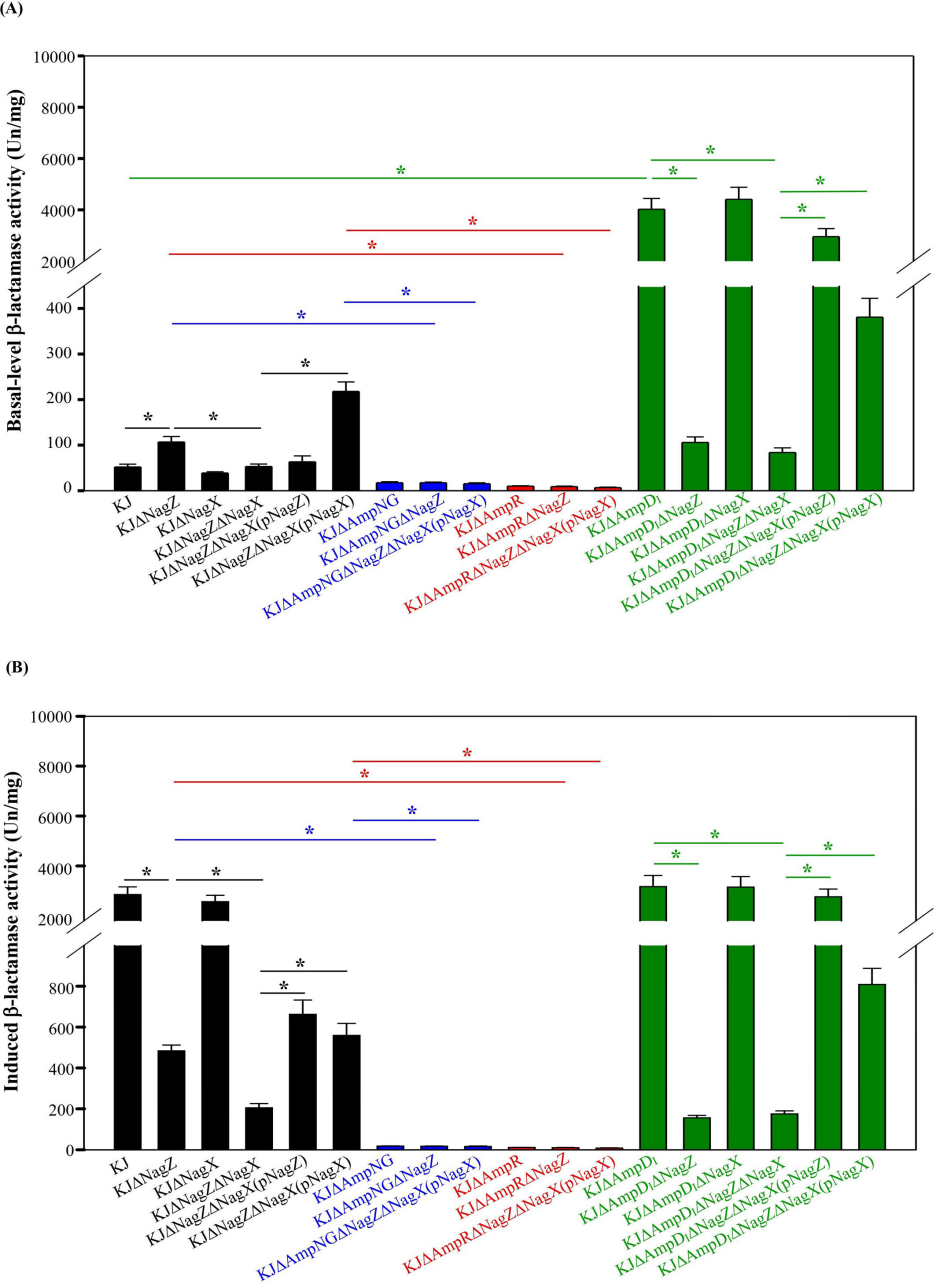
Basal and induced β-lactamase activities of wild-type KJ and its derived strains. Cells were grown overnight, reinoculated into 5 mL fresh LB at an initial OD_450nm_ of 0.15, and cultured for 3 h before treatment without (**A**) or with (**B**) 50 µg/mL CAZ for 30 min. Intracellular β-lactamase activity was assayed using nitrocefin. One unit (Un) was defined as the amount of enzyme hydrolyzing 1 nmol of nitrocefin per minute. Specific activity (Un/mg) was expressed as nanomoles of nitrocefin hydrolyzed per minute per milligram protein. Error bars represent the standard errors of means. *, *P* < 0.05, significance calculated by Student’s *t* test.

We examined whether NagZ-independent β-lactamase expression occurs in *S. maltophilia* KJ by measuring the basal and ceftazidime (CAZ)-induced β-lactamase activities of wild-type KJ and the *nagZ* mutant (KJΔNagZ). Compared to wild-type KJ, KJΔNagZ showed an elevated basal β-lactamase activity (approximately twofold) (Fig. 2A). Upon CAZ challenge, the β-lactamase activity of KJ increased 54-fold, whereas this induction was markedly reduced in KJΔNagZ (Fig. 2B), consistent with the established role of NagZ as the main contributor to β-lactamase induction (24). However, KJΔNagZ still displayed a CAZ-induced β-lactamase activity as high as 484 ± 28 Un/mg, which was significantly higher than the basal activity of wild-type KJ (52 ± 6.3 Un/mg). Taken together, both basal and CAZ-induced β-lactamase activities in KJΔNagZ indicate the presence of NagZ-independent β-lactamase expression.

### *nagZ* mutant displayed detectable β-*N*-acetylglucosaminidase activity

**Fig 3:**
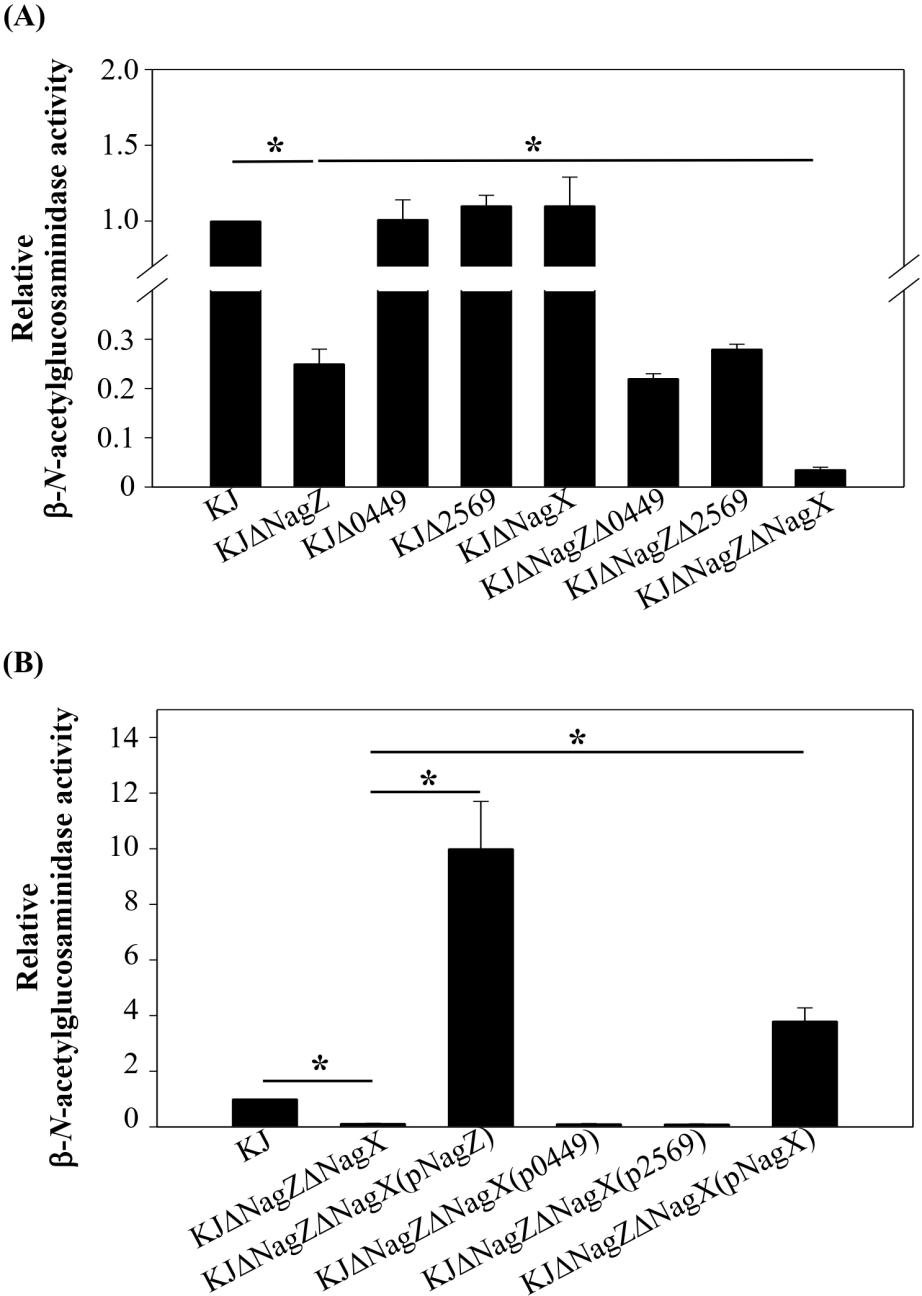
Beta-*N*-acetylglucosaminidase activities of wild-type KJ and its derived strains. Logarithmically grown cells were harvested, and whole cells were subjected to the determination of β-*N*-acetylglucosaminidase activity using *p*-nitrophenyl-β-*N*-acetyl-D-glucosaminide as a substrate. Relative activity was normalized to the wild-type KJ. Bars represent means of three independent experiments. Error bars represent the standard errors of means. *, *P* < 0.05, significance calculated by Student’s *t* test. (**A**) Effect of deleting *smlt0449*, *smlt2569*, and *nagX* on β-*N*-acetylglucosaminidase activity. (**B**) Effect of overexpressing *nagZ*, *smlt0449*, *smlt2569*, and *nagX* on β-*N*-acetylglucosaminidase activity.

NagZ hydrolyzes the glycosidic bonds in GlcNAc-anhMurNAc-peptide, releasing anhMurNAc-peptide, which serves as an AL for β-lactamase expression (30). Considering possible functional redundancy, we asked whether another enzyme with β-*N*-acetylglucosaminidase activity exists in *S. maltophilia*. We therefore measured β-*N*-acetylglucosaminidase activities in wild-type KJ and KJΔNagZ. Compared to wild-type KJ, KJΔNagZ showed a significant reduction in β-*N*-acetylglucosaminidase activity. Nevertheless, residual activity was still detectable in KJΔNagZ (Fig. 3A), indicating that NagZ is not the sole enzyme with β-*N*-acetylglucosaminidase activity in *S. maltophilia*.

### Smlt4027 (NagX), a periplasmic enzyme, harbors β-*N*-acetylglucosaminidase activity

To identify the potential candidates with β-*N*-acetylglucosaminidase activity, we used NagZ as a query to search its homologs in the *S. maltophilia* K279a genome. Two homologs were found: Smlt0449, a cytoplasmic protein annotated as β-glucosidase, and Smlt2569, an 862-aa periplasmic protein annotated as 1,4-β-glucosidase. Smlt0449 and Smlt2569 shared 39%/49% and 29%/47% identity/similarity with NagZ, respectively. In addition, a genome-wide survey revealed that Smlt4027 is annotated as β-*N*-acetylhexosaminidase. β-*N*-acetylhexosaminidase, a member of the family glycoside hydrolases 20 (GH20), acts on *N*-acetylated carbohydrates or glycoproteins (31)

Thus, it is likely that β-*N*-acetylhexosaminidase could hydrolyze the glycosidic bond in GlcNAc-β-1,4-anhMurNAc-peptide. However, Smlt4027 and NagZ showed no significant sequence similarity. Based on our findings in this study, we designated Smlt4027 as NagX. The annotation of *smlt4027* has been deposited in GenBank under accession number PX601588.

To assess whether Smlt0449, Smlt2569, and NagX possess β-*N*-acetylglucosaminidase activities, we constructed in-frame deletion mutants (KJΔ0449, KJΔ2569, and KJΔNagX) and determined their activities. All three mutants exhibited β-*N*-acetylglucosaminidase activities comparable to wild-type KJ (Fig. 3A). Considering that their activities might be masked by NagZ, we introduced *Δsmlt0449*, *Δsmlt2569*, and *ΔnagX* alleles into the KJΔNagZ, respectively. Among the double mutants, only KJΔNagZΔNagX showed reduced β-*N*-acetylglucosaminidase activity compared to KJΔNagZ (Fig. 3A), suggesting that NagX harbors β-*N*-acetylglucosaminidase activity.

Complementation tests were then performed to rule out the possibility of secondary effects that might have resulted from the genetic manipulations of deletion mutant construction. *nagZ*, *smlt0449*, *smlt2569*, or *nagX* were transported into KJΔNagZΔNagX, respectively, and subjected to β-*N*-acetylglucosaminidase activity determination. KJΔNagZΔNagX(pNagZ) and KJΔNagZΔNagX(pNagX), but not KJΔNagZΔNagX(p0449) and KJΔNagZΔNagX(p2569), showed restored activity (Fig. 3B). In summary, *S. maltophilia* harbors at least two β-*N*-acetylglucosaminidases: the cytoplasmic NagZ and the newly identified NagX.

**Fig 4:**
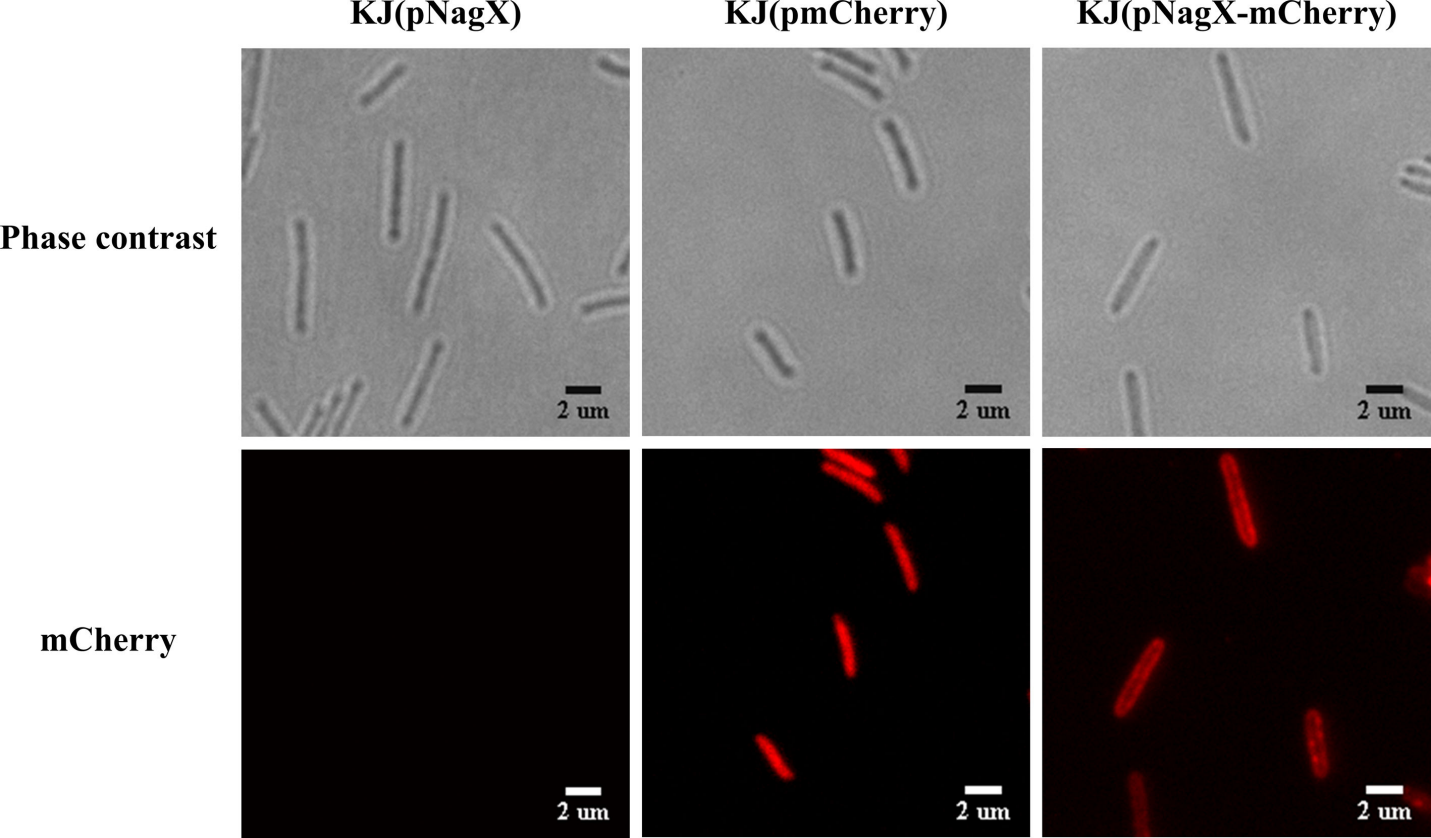
Subcellular localization of NagX in *S. maltophilia* KJ. Logarithmically growing cells of KJ(pNagX), KJ(pmCherry), and KJ(pNagX-mCherry) were fixed with 1.5% paraformaldehyde and immobilized on 1% agarose pads. Localization of the mCherry was visualized using a fluorescence microscope (Leica DM6000B). Top, phase-contrast images; bottom, fluorescence images.

NagX harbors a predicted 29-aa signal peptide (https://services.healthtech.dtu.dk/services/SignalP-6.0/), suggesting that it is a periplasmic protein. To further validate this prediction, we constructed the plasmid pNagX-mCherry, a plasmid with translational fusion of *nagX* and *mCherry* genes, and transported it into wild-type KJ to obtain KJ(pNagX-mCherry). In the meanwhile, we also constructed plasmids containing only *nagX* or mCherry genes, respectively, as control groups. The cellular localization of NagX was confirmed by fluorescence microscopy. KJ(pmCherry) exhibited a uniform-distribution fluorescence signal throughout the cells; however, KJ(pNagX-mCherry) displayed a distinct peripheral fluorescence pattern (Fig. 4). This differential fluorescence distribution supported that NagX is localized in the periplasm.

### NagX contributes to NagZ-independent β-lactamase expression

The role of NagX in NagZ-independent β-lactamase expression was examined by mutant construction and β-lactamase activity assays. KJΔNagX displayed basal and CAZ-induced β-lactamase activities comparable to wild-type KJ (Fig. 2). However, deletion of *nagX* in the *ΔnagZ* background reduced basal β-lactamase and induced β-lactamase activities (KJΔNagZ vs KJΔNagZΔNagX) (Fig. 2). To further compare the individual contributions of NagZ and NagX to β-lactamase activity, basal and induced β-lactamase activities were measured in KJΔNagZΔNagX strains harboring either pNagZ or pNagX. In the *ΔnagZΔnagX* background, ectopic expression of NagX significantly increased both basal and induced β-lactamase activities [KJΔNagZΔNagX vs KJΔNagZΔNagX(pNagX)] (Fig. 2). In contrast, ectopic expression of NagZ affected only induced β-lactamase activity [KJΔNagZΔNagX vs KJΔNagZΔNagX(pNagZ)] (Fig. 2). The basis for this difference is addressed in the Discussion. Collectively, these results indicate that NagX contributes to NagZ-independent β-lactamase expression.

Since KJΔNagZ exhibited higher basal β-lactamase activity than wild-type KJ, and this increase was diminished upon *nagX* inactivation (Fig. 2A), we hypothesized that *nagX* is upregulated when *nagZ* is absent. Quantitative reverse transcription-polymerase chain reaction (qRT-PCR) analysis revealed that *nagX* transcript levels in KJΔNagZ were increased 2.8 ± 0.2-fold compared to wild-type KJ (Fig.S1). Together, these results indicate that loss of cytoplasmic NagZ upregulates periplasmic *nagX* expression, and NagX contributes to NagZ-independent β-lactamase expression.

### Roles of AmpNG, AmpR, and AmpD_I_ in NagX-mediated β-lactamase expression

Since NagX is a periplasmic enzyme with β-*N*-acetylglucosaminidase activity, the processed muropeptides should be transported into the cytosol to serve as ALs for β-lactamase expression. The AmpNG permease system, a known inner-member transporter of muropeptides (20), was the first candidate. Deletion of *ampNG* in KJΔNagZ drastically reduced both basal and CAZ-induced β-lactamase activities (Fig. 2), suggesting that AmpNG is essential for NagX-mediated β-lactamase expression. To confirm this, a *nagX*-expressing plasmid (pNagX) was transported into KJΔNagZΔNagX and KJΔAmpNGΔNagZΔNagX. While KJΔNagZΔNagX(pNagX) showed increased basal and induced β-lactamase activities, this effect was abolished in KJΔAmpNGΔNagZΔNagX(pNagX) (Fig. 2). Thus, AmpNG is the key transporter for the NagX-mediated β-lactamase expression.

AmpR is a well-known transcriptional regulator for NagZ-dependent β-lactamase expression (19). Its role in NagX-mediated β-lactamase expression was also tested. Deletion of *ampR* in KJΔNagZ significantly reduced both basal and CAZ-induced β-lactamase activities. Moreover, although *nagX* overexpression in KJΔNagZΔNagX enhanced basal and induced β-lactamase activities, these were nearly undetectable in KJΔAmpRΔNagZΔNagX(pNagX) (Fig. 2). Therefore, AmpR is indispensable for NagX-mediated β-lactamase expression.

Inactivation of AmpD_I_ leads to derepression of L1/L2 expression even in the absence of β-lactam challenge (21). To elucidate the respective roles of *nagZ* and *nagX* in *ΔampD_I_*-mediated β-lactamase expression, *ΔnagZ* and *ΔnagX* alleles were introduced, either individually or in combination, into the KJΔAmpD_I_ background, generating KJΔAmpD_I_ΔNagZ, KJΔAmpD_I_ΔNagX, and KJΔAmpD_I_ΔNagZΔNagX. These mutants were subsequently examined for basal and induced β-lactamase activities. Compared with KJΔAmpD_I_, both KJΔAmpD_I_ΔNagZ and KJΔAmpD_I_ΔNagZΔNagX exhibited reduced basal and induced β-lactamase activities; in contrast, KJΔAmpD_I_ΔNagX maintained β-lactamase activities comparable to those of KJΔAmpD_I_ (Fig. 2). Furthermore, complementation of KJΔAmpD_I_ΔNagZΔNagX with either *nagZ* or *nagX* restored β-lactamase activity to different extents. Complementation with *nagZ* almost fully restored both basal and induced β-lactamase activities to levels observed in KJΔAmpD_I_, whereas complementation with *nagX* resulted in only partial restoration (Fig. 2).

### NagX contributes to β-lactam resistance

Since β-lactamase is the primary factor conferring β-lactam resistance in *S. maltophilia* (14), we next evaluate the contribution of NagX and NagZ to CAZ susceptibility using E-test. All strains tested for β-lactamase activity in Fig. 2 were included. The trends in CAZ MIC values (Table 1) mirrored those observed for CAZ-induced β-lactamase activities (Fig. 2B). Together, these data indicate that both NagZ and NagX had positive roles in the increase of β-lactamase activity and β-lactam resistance, more significant for NagZ.

**TABLE 1:** Ceftazidime susceptibility of *S. maltophilia* KJ and its derived mutants

Strain	Ceftazidime MIC (μg/mL)
KJ	256
KJΔNagZ	1.5
KJΔNagX	128
KJΔNagZΔNagX	1
KJΔNagZΔNagX(pNagZ)	6
KJΔNagZΔNagX(pNagX)	4
KJΔΑmpDI	>256
KJΔΑmpDIΔNagZ	1
KJΔΑmpDIΔNagX	>256
KJΔΑmpDIΔNagZΔNagX	1
KJΔΑmpDIΔNagZΔNagX(pNagZ)	256
KJΔΑmpDIΔNagZΔNagX(pNagX)	8
KJΔΑmpNG	0.75
KJΔΑmpNGΔNagZ	0.75
KJΔΑmpNGΔNagZΔNagX(pNagX)	0.75
KJΔΑmpR	0.5
KJΔΑmpRΔNagZ	0.5
KJΔΑmpRΔNagZΔNagX(pNagX)	0.5

### The structure of NagX-processed and AmpNG-imported muropeptides

In cells lacking a periplasmic enzyme involved in murein recycling or an inner membrane transporter involved in moving the enzyme-processed products, the substrates of transporter or enzyme should be accumulated in the periplasm or secreted extracellularly. Since NagX-mediated β-lactamase expression was AmpNG-dependent (Fig. 2), we hypothesized that NagX-processed muropeptides are transported by AmpNG. Thus, we compared periplasmic muropeptide profiles of KJ and KJΔAmpNG to clarify AmpNG substrates. Similarly, comparing KJΔAmpNG and KJΔAmpNGΔNagX could reveal NagX substrates and products.

**Fig 5:**
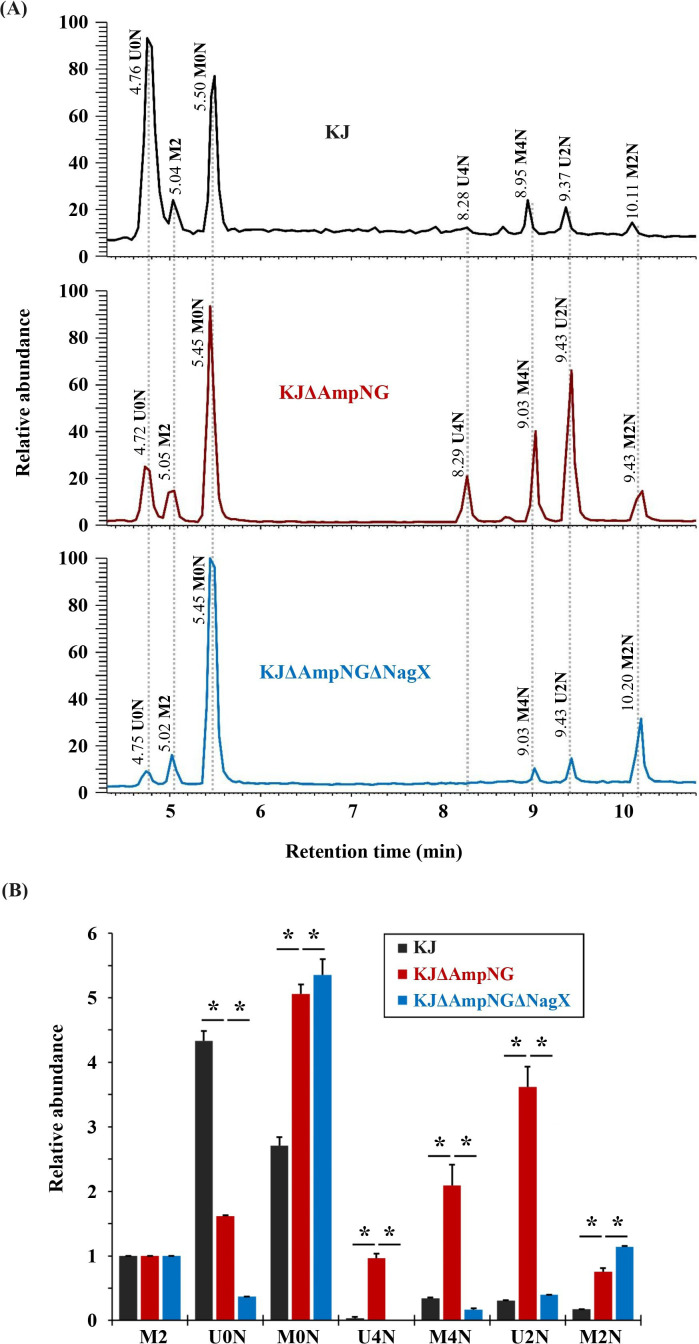
Quantitative LC-MS analysis of periplasmic muropeptides from wild-type KJ, KJΔAmpNG, and KJΔAmpNGΔNagX. Periplasmic muropeptides were extracted by osmotic shock from the logarithmic phase cultures. Purified GlcNAc-MurNAc-dipeptide (M2) was spiked as an internal standard for quantitative LC-MS. (**A**) Extracted ion chromatograms of wild-type KJ (black), KJΔAmpNG (red), and KJΔAmpNGΔNagX (blue) were obtained by reversed-phase ultraperformance liquid chromatography coupled to Orbitrap mass spectrometry. Monitored *m/z* ranges: 698.79–699.79 for [M2+H]^+^ (M2: GlcNAc-MurNAc-dipeptide); 275.61–276.61 for [U0N+H]^+^ (U0N: anhMurNAc); 478.69–479.69 for [M0N+H]^+^ (M0N: GlcNAc-anhMurNAc); 718.81–719.81 for [U4N+H]^+^ (U4N: anhMurNAc-tetrapeptide); 921.89–922.89 for [M4N+H]^+^ (M4N: GlcNAc-anhMurNAc-tetrapeptide); 475.69–476.69 for [U2N+H]^+^ (U2N: anhMurNAc-dipeptide); and 678.77–679.77 for [M2N+H]^+^ (M2N: GlcNAc-anhMurNAc-dipeptide). (**B**) Relative abundance of periplasmic muropeptides in wild-type KJ, KJΔAmpNG, and KJΔAmpNGΔNagX, normalized to M2 (set as 1). A full list of periplasmic muropeptides is provided in Table S3. Data represent the mean of two technical replicates. Error bars indicate the standard error of the mean (SEM). *P < 0.05, significance calculated by Student’s *t* test. Novel muropeptides were further validated by MS/MS (Fig S2).

Periplasmic muropeptides from KJ, KJΔAmpNG, and KJΔAmpNGΔNagX were analyzed by HPLC-MS/MS. Compared to wild-type KJ, KJΔAmpNG accumulated GlcNAc-anhMurNAc (M0N), anhMurNAc-tetrapeptide (U4N), GlcNAc-anhMurNAc-tetrapeptide (M4N), anhMurNAc-dipeptide (U2N), and GlcNAc-anhMurNAc-dipeptide (M2N) (Fig. 5). Comparison of KJΔAmpNG and KJΔAmpNGΔNagX revealed that the levels of M0N were increased, whereas those of U0N were decreased in KJΔAmpNGΔNagX (Fig. 5). A similar trend was observed for M2N and U2N, where elevated M2N and reduced U2N levels in KJΔAmpNGΔNagX (Fig. 5). In contrast, for M4N and U4N, M4N levels were higher in KJΔAmpNG than in KJΔAmpNGΔNagX, while U4N was nearly undetectable in KJΔAmpNGΔNagX (Fig. 5).

## DISCUSSION

NagZ-independent β-lactamase expression has been observed in some microorganisms and appears to be condition-dependent. For example, AmpC expression induced by cefoxitin is NagZ-independent, whereas that induced by cefotaxime is NagZ-dependent in *E. cloacae* complex (29). In *S. maltophilia*, *ΔmrcA*-mediated derepression of basal-level β-lactamase expression is NagZ-independent, but *ΔampD_I_*-mediated expression is largely NagZ-dependent (22). Therefore, NagZ-independent *β*-lactamase expression depends on both genetic background and the type of *β*-lactam. In our previous studies, we showed that NagZ-independent *β*-lactamase expression occurs in response to aztreonam, cefoxitin, carbenicillin, cefuroxime, and piperacillin (22). In this study, we further revealed that NagZ-independent β-lactamase expression is also observed in basal-level and CAZ-induced β-lactamase expression in *S. maltophilia*, and that a periplasmic *N*-acetylglucosaminidase, NagX, contributes to this circuit.

During physiological PG recycling, the LT-processed muropeptide is mainly M4N, which is transported into the cytoplasm via AmpG in *Escherichia coli* (32). Thus, the major periplasmic anhydromuropeptides that accumulate in *ampG* mutant are M4N. Interestingly, in this study we found not only M4N but also M2N, U2N, and U4N in the periplasm of the *ampNG* mutant of *S. maltophilia* (Fig. 5). To our knowledge, this is the first report describing anhydromuropeptides with a two-amino acid stem. The presence of M2N and U2N in the periplasm strongly suggests that an unidentified periplasmic peptidase is involved in processing the M4N stem peptide in *S. maltophilia*.

The periplasmic muropeptide profile assay further showed that M0N and U0N were detected in all strains tested (Fig. 4), suggesting that there is at least one active periplasmic amidase capable of cleaving the amide bond between anhMurNAc and L-alanine. Similar to the cytoplasmic AmpD_I_ and NagZ, the unidentified periplasmic amidase and NagX may also share the same substrates M2N and M4N.

In the known L1/L2 induction model, M4N is the common substrate of AmpD_I_ and NagZ; however, muropeptides processed by AmpD_I_ and NagZ exert distinct effects on L1/L2 expression. That is, products generated by NagZ induced L1/L2 expression, whereas those derived from AmpD_I_ do not (13). In our assay system, we found that the contribution of NagZ to β-lactamase activity was significantly attenuated when AmpD_I_ is functional [KJΔNagZΔNagX(pNagZ) vs KJΔAmpD_I_ΔNagZΔNagX(pNagZ) in Fig. 2], indicating that AmpD_I_ is superior to NagZ in hydrolyzing M4N. Nevertheless, when the β-lactamase activities of KJΔNagZΔNagX(pNagX) and KJΔAmpD_I_ΔNagZΔNagX(pNagX) were compared (Fig. 2), we noticed that the effect of AmpD_I_ on NagX-mediated β-lactamase expression is less profound than that on the NagZ-mediated expression, suggesting that AmpD_I_ is less able to hydrolyze NagX-processed products, such as U4N, U2N, or U0N. Collectively, the cellular location and hydrolytic dominance of NagX, NagZ, and AmpD_I_ determine the degree of L1/L2 expression. This may be the reason why KJΔNagX and KJ have comparable basal and induced β-lactamase activities (Fig. 2) because cytoplasmic NagZ and AmpD_I_ override the contribution of NagX to β-lactamase expression in wild-type KJ.

To clarify the substrates of NagX, we compared the periplasmic muropeptide profile between KJΔAmpNG and KJΔAmpNGΔNagX. Compared with KJΔAmpNG, M0N and M2N increased, whereas U0N and U2N decreased in KJΔAmpNGΔNagX (Fig. 5). Furthermore, NagX exhibited β-*N*-acetylglucosaminidase activity (Fig. 3). Together, these results indicate that M0N and M2N are substrates of NagX, which processes M0N and M2N into U0N and U2N, respectively. Based on this understanding of NagX, it is unexpected that M4N levels in KJΔAmpNGΔNagX were lower than in KJΔAmpNG (Fig. 5). This reduction may result from processing M4N into M2N or M0N by unidentified peptidases or amidases in KJΔAmpNGΔNagX.

NagZ and NagX are β-*N*-acetylhexosaminidases (EC number 3.2.1.52), generally functioning as exo-enzymes that catalyze the cleavage of terminal β-d-GlcNAc and β-d-GalNAc residues in *N*-acetylhexosaminides (33). *N*-acetylhexosaminidases have diverse functions depending on the microorganism and their cellular localization. Cytoplasmic NagZ belongs to GH3 family, while periplasmic NagX is classified as GH20. Conserved motifs for GH3 and GH20 β-*N*-acetylhexosaminidases have been proposed: “KH(F/I)PG(H/L)GX(4)D(S/T)H” for GH3 (34) and “(H/N)-X-G-(A/C/G/M)-D-E-(A/I/L/V)” for GH20 (33). These motifs are found in residues 163–176 of NagZ and residues 346–352 of NagX, respectively. Based on known functions, cytoplasmic GH3 β-*N*-acetylhexosaminidases hydrolyze the β-1,4-glycosidic bond between GlcNAc and 1,6-anhMurNAc, linking them to PG recycling and β-lactamase expression (35). In contrast, periplasmic GH20 β-*N*-acetylhexosaminidases have mainly been reported to participate in chitin degradation and the generation of chitinase inducers, as seen in *Vibrio furnissii*, *Alteromonas* sp. *Cellvibrio japoicus*, and *Aeromonas hydrophilia* (36–39). In this study, we identified NagX as a novel periplasmic GH20 β-*N*-acetylhexosaminidase contributing to β-lactamase expression—this is the first report of such a role. Although NagZ and NagX have overlapping functions, their protein sequences share little similarity.

It has long been accepted that anhMurNAc-peptides serve as ALs for β-lactamase expression in microorganisms harboring chromosomally encoded AmpR-β-lactamase systems (40). In *P. aeruginosa*, ALs responsible for AmpC expression have been extensively studied: U3N for *ampD* mutant, and U5N and U3N for the *dacB* mutant (41). Using AmpC expression in *P. aeruginosa* as a model, we reason that the periplasmic M4N, M2N, U4N, and U2N could serve as ALs precursors (M4N and M2N) or ALs (U4N and U2N) for L1/L2 expression.

*E. coli* AmpG exhibits a strict substrate specificity, transporting only muropeptides containing anhydro-disaccharide moiety (M5N, M4N, M3N, and M0N), but not GlcNAc-MurNAc-peptides or anhMurNAc (32). By comparing the muropeptides profiles of KJ and KJΔAmpNG (Fig. 5), we inferred that the muropeptides accumulated in KJΔAmpNG represent substrates of the AmpNG permease. Based on this comparison, AmpNG was concluded to transport U4N and U2N, in addition to M4N, M2N, and M0N (Fig. 5). In contrast, the level of U0N was not elevated in KJΔAmpNG relative to that in wild-type KJ, suggesting that U0N is not a compatible substrate for AmpNG. Notably, the relative abundance of U0N in wild-type KJ was considerably higher than that of U4N and U2N (Fig. 5), further supporting the conclusion that U0N is not transported by AmpNG. There should be an unidentified inner membrane transporter for U0N transportation from the periplasm to the cytosol and this transporter is more efficient in the *ampNG* mutant. Herein, we propose two possible explanations for the differences in substrate specificity between *E. coli* AmpG and *S. maltophilia* AmpNG: (i) AmpN, as part of the AmpNG permease system, may broaden the substrate specificity; (ii) the evolution of periplasmic muropeptides-hydrolyzing enzymes and inner membrane muropeptide permeases must be coordinated to ensure efficient delivery of degraded murein into the cytoplasm. Notably, no NagX homolog was found in *E. coli* when we queried its genome with *S. maltophilia* NagX. The absence of NagX in *E. coli* may explain why AmpG lacks the ability to transport U4N. By contrast, in *S. maltophilia*, anhMurNAc-peptides are generated in the periplasm via NagX activity, making them natural substrates for AmpNG permease.

Integrating previous findings with novel insights from this study, we propose a model for L1/L2 induction in *S. maltophilia* (Fig. 1). During PG turnover, PG is primarily cleaved by LT to generate M4N. M4N can then be hydrolyzed by periplasmic NagX and/or unidentified peptidase and amidase to produce GlcNAc, U4N, M2N, U2N, M0N, and U0N. M4N, M2N, M0N, U4N, and U2N are transported into the cytoplasm via the AmpNG permease. In the cytoplasm, M4N and M2N can be further hydrolyzed by NagZ, generating U4N and U2N. The U4N and U2N, produced either in the periplasm or cytoplasm, act as ALs for L1/L2 expression. Collectively, these findings refine our understanding by demonstrating that L1/L2 expression is modulated not only by cytoplasmic but also by periplasmic muropeptide-processing enzymes.

## MATERIALS AND METHODS

### Bacterial strains, plasmids, and primers

Bacterial strains and plasmids used in this study are listed in Table S1. Primers used are listed in Table S2.

### β-lactamase activity determination

Bacterial cultures were inoculated into fresh LB with an initial OD_450nm_ of 0.15. After incubation at 37°C for 3 h, cultures were treated with or without 50 μg/mL CAZ for 0.5 h. Cells were harvested, lysed, and subjected to β-lactamase activity assays using nitrocefin (Cyrusbioscience, Taiwan) as the substrate, as described previously (22). One unit of enzyme activity (Un) was defined as the amount of enzyme required to hydrolyze 1 nmole of nitrocefin per min. Specific activity (Un/mg) was expressed as nmole of nitrocefin hydrolyzed per min per mg of protein. Protein concentrations were determined using the Bio-Rad protein assay reagent with bovine serum albumin as the standard. Each assay was performed independently at least three times.

### β-*N*-acetylglucosaminidase activity determination

Bacterial cultures were inoculated into fresh LB broth at an initial OD_450nm_ of 0.15. After incubation at 37°C for 2.5 h, cells were harvested and lysed by sonication. Following centrifugation, the supernatant was collected as the whole-cell lysate. A 500 μL of whole-cell was mixed with 400 μL of reaction buffer containing 1 mM *p*-nitrophenyl*-*β*-N*-acetyl-D-glucosaminide (Sigma-Aldrich) and incubated at room temperature for 5 h. Reactions were terminated by the addition of 100 μL of 2.5 M K_2_CO_3_. After centrifugation, the OD_405nm_ of supernatants was measured. Relative β*-N*-acetylglucosaminidase activity was normalized to that of wild-type KJ. All assays were performed independently at least three times.

### Construction of in-frame deletion mutants

In-frame deletion mutants were constructed using the double cross-over homologous recombination method as described previously (21). DNA fragments upstream and downstream of the target gene were amplified from *S. maltophilia* KJ genomic DNA by PCR and cloned into plasmid pEX18Tc, generating pΔ0449, pΔ2569, and pΔNagX. Primer sets used were Smlt0449N-F/R and Smlt0449C-F/R for pΔ0449, Smlt2569N-F/R and Smlt2569C-F/R for pΔ2569, as well as NagXN-F/R and NagXC-F/R for pΔNagX (Tables S1 and S2). The pEX18Tc-derived plasmids were introduced into *S. maltophilia* KJ via conjugation. Transconjugants were selected on LB agar plates containing 30 μg/mL tetracycline and 2.5 μg/mL norfloxacin. Deletion mutants were then counter-selected on LB agar plates containing 10% sucrose. Correctness of mutants was confirmed by PCR and sequencing.

### Construction of complementation plasmids

The intact *smlt0449*, *smlt2569*, and *nagX* genes were amplified by PCR using primer sets Smlt0449-F/R, Smlt2569-F/R, and NagX-F/R (Table S2), and cloned into plasmid pRK415, yielding p0449, p2569, and pNagX, respectively.

### Quantitative reverse transcription-polymerase chain reaction

DNA-free RNA was prepared from mid-log phase bacterial cells and converted to cDNA using a High-Capacity cDNA Reverse Transcription Kit (Applied Biosystems). qRT-PCR was performed using the StepOnePlus Real-Time PCR System following the manufacturer’s protocol. Gene expression levels were normalized to those of 16S rRNA. Fold changes were calculated using the ΔΔC_T_ method (42*).*

### β-lactam susceptibility test

The CAZ susceptibility of *S. maltophilia* was determined by E-test according to the manufacturer’s guidelines. The endpoint of the E-test was read as the interception of the graded strips with the elliptical zone of inhibition.

### Construction of pmCherry and pNagX-mCherry

To construct the plasmid pmCherry, the intact mCherry gene was amplified from the plasmid TPC2-mCherry (43) with primer sets mCherry-F/R (Table S2), and cloned into plasmid pRK415, yielding pmCherry. For the pNagX-mCherry translational fusion construction, the *nagX* gene without a stop codon was amplified from the chromosome of KJ by PCR using primer sets NagX-fluo-F/R (Table S2), and cloned into plasmid pmCherry, yielding pNagX-mCherry.

### Fluorescence microscopy

Mid-log phase cultures were diluted to an OD_450nm_ of 0.1 and fixed with 1.5% paraformaldehyde. A 1% agarose pad was prepared on a microscope slide, and the fixed cells were immobilized on the pad surface. Phase-contrast and fluorescence images were obtained using a Leica DM6000B motorized upright fluorescence microscope equipped with a 100× oil immersion objective. For mCherry detection, a standard Texas Red (TX2) filter set was used. Images were acquired and analyzed using MetaMorph software. All imaging parameters, including exposure time and gain, were kept constant across samples for comparative analysis.

### Preparation of M2 muropeptides

GlcNAc-MurNAc-dipeptide (M2) was used as the internal standard to quantify periplasmic muropeptides and was prepared from total cell muropeptides as described previously (44). Bacterial cultures were inoculated into 100 mL fresh LB broth and incubated at 37°C for 5 h. After centrifugation at 4,500 rpm for 15 min, pellets were resuspended in 3 mL PBS and added to 6 mL of boiling 6% SDS. The mixtures were boiled and stirred for 2 h, then stirred overnight at room temperature. After ultracentrifugation at 40,000 × *g* for 20 min at room temperature, murein sacculi-containing pellets were washed and resuspended in 900 μL Milli-Q water. To digest Braun’s lipoprotein, the suspension was treated with 200 μg/mL protease (Sigma-Aldrich) in 1 M Tris-HCl buffer (pH 8.0) and incubated at 37°C for 1 h. Reactions were stopped by adding 10% SDS and boiling for 5 min. After cooling, mixtures were diluted with 2 mL Milli-Q water and ultracentrifuged at 40,000 × *g* for 20 min. To digest the murein sacculi, pellets were resuspended in 200 μL 50 mM sodium phosphate buffer (pH 4.9) containing 10 μg/mL lysozyme (Sigma-Aldrich) and incubated at 37°C for 16 h. Reactions were terminated by boiling for 5 min, and mixtures were centrifuged at 20,000 × *g* for 15 min. Supernatants were adjusted to pH 8.5–9.0 with borate buffer, and the terminal MurNAc residues were reduced to NAc-muraminitol using 2 M NaBH_4_ for 30 min. Samples were adjusted to pH 3–4 with 50% orthophosphoric acid.

### Purification of M2 muropeptides

Total cell muropeptides were centrifuged at 14,000 × *g* for 30 min at 4°C, and the resulting supernatant was subjected to purification by liquid chromatography using an Agilent 1260 Infinity LC system. Soluble muropeptides were separated on a SunFire C18 column (100 Å, 5 µm, 4.6 × 250 mm; Waters), with solvent A (0.1% trifluoroacetic acid in water) and solvent B (0.1% trifluoroacetic acid in acetonitrile) as mobile phases. The column was pre-equilibrated with 2% solvent B at a flow rate of 1.0 mL/min before injection. Each run used 100 μL of sample, with three replicate injections performed. A 60-min gradient was applied for elution as follows: 0–1 min, 2%–5% B (linear); 1–6 min, 5% B; 6–9 min, 5%–8% B (linear); 9–29 min, 8%–15.5% B (linear); 29–39 min, 15.5%–95% B (linear); 39–49 min, 95% B; 49–50 min, 95%–2% B (linear); and 50–60 min, 2% B. Fractions were collected every 30 s. The purity of each fraction was confirmed by a hybrid LTQ-Orbitrap Elite mass spectrometer (Thermo Scientific, USA). Fractions containing M2 were pooled, dried with an Eppendorf Vacufuge Plus concentrator, reconstituted in 30 μL ultrapure water, and stored at −80°C for later use as an internal standard in periplasmic muropeptide analysis. Prior to the analysis, the signal intensity of the reconstituted M2 was evaluated, and an equal amount of M2 was spiked into all samples, as M2 is not endogenously present in periplasmic muropeptides. Relative quantification of periplasmic muropeptides was performed by internal standard normalization, with M2 used as the reference compound.

### Preparation of periplasmic muropeptides

Periplasmic lysates containing soluble muropeptides were prepared using an osmotic shock method (44). The supernatant was adjusted to pH 8.5–9.0 with borate buffer, followed by the reduction of the terminal MurNAc to NAc-muraminitol using 2 M NaBH_4_ for 30 min. Samples were adjusted to pH 3–4 with 50% orthophosphoric acid prior to LC-MS analysis.

### LC-MS analysis of periplasmic muropeptides

Periplasmic muropeptide samples were centrifuged at 14,000 × *g* for 30 min at 4°C. Twenty-nine microliters of each supernatant was mixed with 1 μL of purified M2 internal standard and subjected to LC-MS analysis using a Vanquish UPLC system (Thermo Scientific, USA) coupled to a hybrid LTQ-Orbitrap Elite mass spectrometer. Chromatography separation was performed on an ACQUITY UPLC CSH C18 column (130 Å, 1.7 µm, 2.1 × 100 mm, Waters), with solvent A (0.1% formic acid in water) and solvent B (0.1% formic acid in acetonitrile) as the mobile phases. Prior to injection, the column was pre-equilibrated at 52°C with 2% solvent B at a flow rate of 200 µL/min. The injection volume was 10 μL, with duplicate injections performed. A 24-min gradient was applied for elution as follows: 0–0.7 min, 2% B; 0.7–2.5 min, 2%–5% B (linear); 2.5–4 min, 5% B; 4–6 min, 5%–8% B (linear); 6–11 min, 8%–9.5% B (linear); 11–12.5 min, 9.5%–11% B (linear); 12.5–14 min, 11%–90% B (linear); 14–17 min, 90% B; 17–17.5 min, 90%–2% B (linear); and 17.5–24 min, 2% B. Detection was carried out using a heated electrospray ionization (HESI) source in positive ionization mode with the following settings: spray voltage, 3.5 kV; capillary temperature, 320°C; HESI heater temperature, 300°C; sheath gas flow, 40 (A.U.); and auxiliary gas, 12 (A.U.). Data were acquired in top-5 data-dependent acquisition (DDA) mode. Each cycle consisted of one full Fourier Transform mass spectrometry (FT-MS) scan (*m/z* range of 200–2,000 at a resolution of 60,000), followed by five DDA MS/MS scans (resolution 15,000). MS/MS spectra were acquired using collision-induced dissociation with default charge state of 1, an isolation width of 2.0 *m/z*, a normalized collision energy of 25.0, activation Q of 0.250, and an activation time of 10.0 ms. Maximum injection time was 500 ms for both full FT-MS and MS/MS, with automatic gain control targets of 3.00 × 10^6^ and 3.00 × 10^4^, respectively. All mass spectra were recorded in profile mode.

### LC-MS data processing

Raw LC-MS/MS data were converted to an mzXML format using MSConvert (Proteo Wizard) and analyzed with the high-throughput automated muropeptide analysis (HAMA) software (version 4.0106) for mass deconvolution and peptidoglycan identification as previously described (45). HAMA integrates three modules—DBuilder, Analyzer, and Viewer—for database construction, data analysis, and visualization of chromatograms and annotated spectra.

A species-specific muropeptide database was generated *in silico*, including all possible combinations of GlcNAc, MurNAc, peptide stems, and corresponding modifications. After loading the *S. maltophilia*-specific database, mzXML-formatted raw file, and user-defined parameters, the Analyzer processed the MS data and output an Excel file listing all identified muropeptides with charge state, molecular weight, retention time, peak intensity, areas under the curve (AUC), sequence, main scan number, cosine similarity, score, and other metrics. Parameters were: MS1 scan range *m/z* 200–2,000; retention time 1.5–14 min; mass tolerance 10 ppm; intensity threshold 1 × 10^4^; MS1 isotopic peaks tolerance 0.01 Da with 30% intensity tolerance; MS2 precursor tolerance 0.5 Da; and MS2 fragments tolerance 0.1 Da. AUCs of identical muropeptides eluting at the same retention time—including variants with different adduct ions, charge states, or in-source fragmentation products—were summed. Relative abundance was determined by normalizing the AUC of each muropeptide to that of the internal standard M2, allowing for inter-sample comparison. All LC-MS identifications were manually inspected to confirm the performance of the automatic identification pipeline.

## SUPPLEMENTAL MATERIAL

Supplemental material aac.00243-26-s0001.docx Tables S1 to S3; Fig. S1 and S2.

ASM does not own the copyrights to Supplemental Material that may be linked to, or accessed through, an article. The authors have granted ASM a non-exclusive, world-wide license to publish the Supplemental Material files. Please contact the corresponding author directly for reuse.
