# Supplementary material for: NagX, a periplasmic β-N-acetylglucosaminidase, contributes to NagZ-independent β-lactamase expression in Stenotrophomonas maltophilia
Source: Antimicrob Agents Chemother. 2026 Jul 16;70(8):e00243-26. doi: 10.1128/aac.00243-26 (PMC13436218; doi:10.1128/aac.00243-26)
Supplement: Supplementary file 1 [file aac.00243-26-s0001.docx]

**Table S1 Bacterial strains and plasmids used in this study**

| Strain or plasmid | Genotype or properties | Reference |
| --- | --- | --- |
| ***S. maltophilia***  KJ  KJ∆NagZ  KJ∆0449  KJ∆2569  KJ∆NagX  KJ∆NagZ∆0449  KJ∆NagZ∆2569  KJ∆NagZ∆NagX  KJ∆AmpNG  KJ∆AmpNG∆NagZ  KJ∆AmpNG∆NagX  KJ∆AmpNG∆NagZ  ΔNagX  KJΔAmpR  KJ∆AmpR∆NagZ  KJ∆AmpR∆NagZ  ΔNagX  KJΔAmpD_I_  KJΔAmpD_I_ΔNagZ  KJΔAmpD_I_ΔNagX  KJAmpD_I_ΔNagZ  ΔNagX | A clinical *S. maltophilia* isolate  *S. maltophilia* KJ mutant of *nagZ* gene; *ΔnagZ*  *S. maltophilia* KJ mutant of *smlt0449* gene; *Δ0449*  *S. maltophilia* KJ mutant of *smlt2569* gene; *Δ2569*  *S. maltophilia* KJ mutant of *nagX* gene; *ΔnagX*  *S. maltophilia* KJ mutant of *nagZ* and *smlt0449* genes; *ΔnagZ*, *Δ0449*  *S. maltophilia* KJ mutant of *nagZ* and *smlt2569* genes; *ΔnagZ*, *Δ2569*  *S. maltophilia* KJ mutant of *nagZ* and *nagX* genes; *ΔnagZ*, *ΔnagX*  *S. maltophilia* KJ mutant of *ampN* and *ampG* genes; *ΔampN*, *ΔampG*  *S. maltophilia* KJ mutant of *ampN*, *ampG,* and *nagZ* genes; *ΔampN*, *ΔampG*, *ΔnagZ*  *S. maltophilia* KJ mutant of *ampN*, *ampG*, and *nagX* genes; *ΔampN*, *ΔampG*, *ΔnagX*  *S. maltophilia* KJ mutant of *ampN*, *ampG*, *nagZ*, and *nagX* genes; *ΔampN*, *ΔampG*, *ΔnagZ*, *ΔnagX*  *S. maltophilia* KJ mutant of *ampR* gene; *ΔampR*  *S. maltophilia* KJ mutant of *ampR* and *nagZ* genes; *ΔampR*, *ΔnagZ*  *S. maltophilia* KJ mutant of *ampR*, *nagZ*, and *nagX* genes; *ΔampR*, *ΔnagZ*, *ΔnagX*  *S. maltophilia* KJ mutant of *ampD_I_* gene; *ΔampD_I_*  *S. maltophilia* KJ mutant of *ampD_I_* and *nagZ* genes; *ΔampD_I_*, *ΔnagZ*  *S. maltophilia* KJ mutant of *ampD_I_* and *nagX* genes; *ΔampD_I_*, *ΔnagX*  *S. maltophilia* KJ mutant of *ampD_I_*, *nagZ*, *and nagX* genes; *ΔampD_I_*, *ΔnagZ*, *ΔnagX* | 1  2  This study  This study  This study  This study  This study  This study  3  This study  This study  This study  3  This study  This study  4  2  This study  This study |
| ***E. coli***  DH5α  S17-1 | F- φ80d*lacZΔM15* Δ(*lacZYA-argF*)*U169* *deoR recA1 endA1 hsdR17* (r_k_^-^ m_k_^+^) *phoA supE44λ* *thi-1 gyrA96 relA1*  λ*pir*^+^ mating strain | Invitrogen  5 |
| **Plasmids** |  |  |
| pEX18Tc | *sacB oriT*; Tc^r^ | 6 |
| pRK415 | Mobilizable broad-host-range plasmid cloning vector, RK2 origin; Tc^r^ | 7 |
| p∆NagZ  p∆0449  p∆2569  p∆NagX  p∆AmpNG  p∆AmpR  pNagZ  p0449  p2569  pNagX  TPC2-mCherry  pmCherry  pNagX-mCherry | pEX18Tc with an internal-deletion *nagZ* gene; Tc^r^  pEX18Tc with an internal-deletion *smlt0449* gene; Tc^r^  pEX18Tc with an internal-deletion *smlt2569* gene; Tc^r^  pEX18Tc with an internal-deletion *nagX* gene; Tc^r^  pEX18Tc with an internal-deletion *ampN* and *ampG* gene; Tc^r^  pEX18Tc with an internal-deletion *apmR* gene; Tc^r^  pRK415 with an intact *nagZ* gene; Tc^r^  pRK415 with an intact *smlt0449* gene; Tc^r^  pRK415 with an intact *smlt2569* gene; Tc^r^  pRK415 with an intact *nagX* gene; Tc^r^  *Tpcn2 mCherry pUC/ColE1*; Amp^r^  pRK415 with an intact *mCherry* gene; Tc^r^  pRK415 with *nagX-mCherry* translational fusion; Tc^r^ | 2  This study  This study  This study  3  3  2  This study  This study  This study  8  This study  This study |

1. Hu RM, Huang KJ, Wu LT, Hsiao YJ, Yang TC. Induction of L1 and L2 β-lactamases of *Stenotrophomonas maltophilia*. *Antimicrob Agents Chemother*. 2008;52(3):1198-200.
2. Huang YW, Hu RM, Lin CW, Chung TC, Yang TC. NagZ-dependent and NagZ-independent mechanisms for β-lactamase expression in *Stenotrophomonas maltophilia*. *Antimicrob Agents Chemother*. 2015;56(4):1936-41.
3. Lin CW, Lin HC, Huang YW, Chung TC, Yang TC. Inactivation of *mrcA* gene derepresses the basal-level expression of L1 and L2 β-lactamases in *Stenotrophomonas maltophilia*. *J Antimicrob Chemother*. 2011;66(9):2033-7.
4. Yang TC, Huang YW, Hu RM, Huang SC, Lin YT. AmpD_I_ is involved in expression of the chromosomal L1 and L2 beta-lactamases of Stenotrophomonas maltophilia. *Antimicrob Agents Chemother*. 2009;53(7):2902-2907.
5. Simon R, O'Connell M, Labes M, Puhler A. Plasmid vector for the genetic analysis and manipulation of *Rhizobia* and other Gram-negative bacteria. *Methods Enzymol*. 1986;118:640-59.
6. Hoang TT, Karkhoff-Schweizer RR, Kutchma AJ, Schweizer HP. A broad-host-range Flp-FRT recombination system for site-specific excision of chromosomally-located DNA sequences: application for isolation of unmarked *Pseudomonas aeruginosa* mutants. *Gene*. 1998;212:77-86.
7. Keen NT, Tamaki S, Kobayashi D, Trollinger D. Improved broad-host-range plasmids for DNA cloning in gram-negative bacteria. *Gene*. 1998*;*70:191-7.
8. Ruas M, Davis LC, Chen CC, et al. Expression of Ca^2+^-permeable two-pore channels rescues NAADP signalling in TPC-deficient cells. *EMBO J*. 2015;34(13):1743-1758.

**Table S2 Primers used in this study**

| primer | Sequence (5’→3’) | Purpose |
| --- | --- | --- |
| Smlt0449N-F  Smlt0449N-R  Smlt0449C-F  Smlt0449C-R | GGGAATTCATACCGACAT  ACACTCTAGAACAGCGAG  ACTTCTAGAACCACCCGC  CCCAAGCTTCCCCTTTTT | pΔ0449 construction |
| Smlt2569N-F  Smlt2569N-R  Smlt2569C-F  Smlt2569C-R | TCCGAAGCTTGTCCGTCATTCC  CCCTGCAGGCCTTCGACGAAG  GGCTGCAGCAACACCGACTGC  TCCGAATTCGTCTCAGCATTCG | pΔ2569 construction |
| NagXN-F  NagXN-R  NagXC-F  NagXC-R | GAAAGCTTACAACGTCGGTGCC  GTAAGCTTGGCAGGAATCAGCATC  TCTCTAGAACTTCCAGCTTGC  TAGAGCTCGTGATCGTGGTC | pΔNagX construction |
| Smlt0449-F  Smlt0449-R | GGCAAGCTTACCGACATGA  CGCGAGCTCCATCGAAAC | p0449 construction |
| Smlt2569-F  Smlt2569-R | GGAAGCTTCGGTTCCGACTGA  CATCTAGACTACTGCGACGCACACT | p2569 construction |
| NagX-F  NagX-R | GCGCTCAAGCTTAACAACCCGACCTA  AGGAATTCGCCATGCGTAGATGCTCTG | pNagX construction |
| mCherry-F  mCherry-R | GGCAAGCTTGGTGAGCAAGG  GCGGAATTCCTACTTGTACAGCTCG | pmCherry construction |
| NagX-fluo-F  NagX-fluo-R | GCCAAGCTTGGGTCATTC  CGGGGTACCTTTCCCCGGGGTCAC | pNagX-mCherry  construction |

**Table S3. Periplasmic muropeptides of wild-type KJ, KJΔAmpNG, and KJΔAmpNGΔNagX analyzed by LC-MS**

| R.T. (min) | Annotation | Charge  state | *m/z* of monoisotope | | Proposed formula | Error (ppm) | Reference |
| --- | --- | --- | --- | --- | --- | --- | --- |
|  |  |  | **Measured** | **Theoretical** |  |  |  |
| 4.75 | U0N | 1 | 276.1074 | 276.1083 | C_11_H_18_O_7_N_1_^+^ | 3.46 |  |
|  |  | 1 | 298.0895 | 298.0903 | C_11_H_17_O_7_N_1_Na^+^ | 2.64 |  |
| 5.03 | M2 | 1 | 699.2926 | 699.2936 | C_27_H_47_O_17_N_4_^+^ | 1.49 | 1 |
|  |  | 1 | 721.2763 | 721.2756 | C_27_H_47_O_17_N_4_Na^+^ | -1.08 |  |
|  |  | 1 | 496.2133 | 496.2143 | C_19_H_34_O_12_N_3_^+^ | 1.99 |  |
| 5.45 | M0N | 1 | 479.1877 | 479.1877 | C_19_H_31_O_12_N_2_^+^ | -0.04 |  |
|  |  | 1 | 501.1689 | 501.1696 | C_19_H_30_O_12_N_2_Na^+^ | 1.59 |  |
| 8.23 | U4N | 1 | 719.3091 | 719.3099 | C_29_H_47_O_15_N_6_^+^ | 1.15 | This study |
| 8.98 | M4N | 1 | 922.3884 | 922.3893 | C_37_H_60_O_20_N_7_^+^ | 1.02 | 2 |
|  |  | 2 | 461.6978 | 461.6986 | C_37_H_61_O_20_N_7_^2+^ | 1.74 |  |
|  |  | 1 | 719.3091 | 719.3099 | C_29_H_47_O_15_N_6_^+^ | 1.14 |  |
| 9.39 | U2N | 1 | 476.1873 | 476.1880 | C_19_H_30_O_11_N_3_^+^ | 1.46 | This study |
|  |  | 1 | 498.1690 | 498.1700 | C_19_H_29_O_11_N_3_Na^+^ | 2.05 |  |
| 10.15 | M2N | 1 | 679.2660 | 679.2674 | C_27_H_43_O_16_N_4_^+^ | 2.04 | This study |
|  |  | 1 | 701.2494 | 701.2494 | C_27_H_42_O_16_N_4_Na^+^ | -0.05 |  |

1. Kühner D, Stahl M, Demircioglu DD, Bertsche U. From cells to muropeptide structures in 24 h: peptidoglycan mapping by UPLC-MS. Sci Rep. 2014;4:7494.
2. Bernal-Cabas M, Ayala JA, Raivio TL. The Cpx envelope stress response modifies peptidoglycan cross-linking via the L,D-transpeptidase LdtD and the novel protein YgaU. J Bacteriol. 2015;197(3):603–14.

*

**Relative *nagX* transcript**

**KJ	KJΔNagZ**

**Fig. S1. Effects of *nagZ* deletion on the *nagX* transcript level.** Wild-type KJ and KJΔNagZ were inoculated into fresh LB at an initial OD_450_ of 0.15. After 5-h culture, the *nagX* transcript level was determined by qRT-PCR and normalized to 16S rRNA. Fold change is shown relative to KJ cells. Data are mean ± SD of three independent experiments; significance was determined using a two-tailed Student’s *t*-test . *, *P* ≤ 0.05.

**(A)**

**(B)**

**(C)**

**Relative abundance**

**100**

**80**

**60**

**40**

**20**

**0**

**Relative abundance**

**Relative abundance**

**100**

**80**

**60**

**40**

**20**

**0**

**100**

**80**

**60**

**40**

**20**

**0**

**150	200	250	300	350	400	450**

***m/z***

***m/z***

***m/z***

**200 250 300 350 400 450 500 550 600 650**

**200** **250 300 350 400 450 500 550 600 650 700**

**N**

**NAM**

**N**

**N**

**NAM**

**NAM**

**NAG**

**A**

**iE**

**A**

**iE**

**A**

**iE**

**DAP**

**A**

329.1347

148.0607

291.1190

148.0607

291.1190

329.1347

301.1396

237.1078

476.1875

476.1875

291.1186

291.1186

301.1395

329.1344

329.1344

532.2136

532.2136

204.0865

204.0865

458.1771

496.1928

611.2545

455.1930

455.1930

302.1348

302.1348

329.1347

329.1347

630.2619

630.2619

534.2406

534.2406

391.1826

391.1826

262.1398

262.1398

244.1295

427.1822

516.2295

612.2509

701.2990

**Fig. S2. Tandem MS analysis of U2N, M2N, and U4N.** Annotated MS/MS spectra of (A) U2N (*m/z* 476.19, retention time 9.45 min), (B) M2N (*m/z* 679.27, retention time 10.16 min), and (C) U4N (*m/z 7*19.31, retention time 8.30 min).
